# Colonic Polypoid Vascular Ectasia in a Patient With Rectal Prolapse

**DOI:** 10.7759/cureus.26772

**Published:** 2022-07-12

**Authors:** Ryan Meader, Ahmed Khattab, Nahren Asado, Scott Siglin

**Affiliations:** 1 Internal Medicine, Advocate Lutheran General Hospital, Park Ridge, USA; 2 Internal Medicine/Gastroenterology, Advocate Lutheran General Hospital, Park Ridge, USA; 3 Pathology, Advocate Lutheran General Hospital, Park Ridge, USA

**Keywords:** colonoscopy, polypectomy, rectal prolapse, pedunculated polyp, vascular ectasia

## Abstract

Vascular ectasia is a common cause of lower gastrointestinal (GI) bleeding in older patients. They typically present as flat or slightly raised fern-like bright red lesions. We report a rare case of a vascular ectasia presenting as a pedunculated polypoid lesion in a young patient with rectal prolapse. The pedunculated polyp was removed using hot snare polypectomy. This case highlights a unique presentation of a rare lesion and endoscopic management of these lesions.

## Introduction

Colonic vascular ectasia (VE) is not an uncommon cause of lower gastrointestinal (GI) bleeding, especially in the elderly. They typically present as flat or slightly elevated reddish lesions. On the other hand, colonic submucosal VE presenting as pedunculated polypoid lesions are rare. We report an interesting case of colonic vascular ectasia pedunculated polypoid lesion (VEPPL) in a patient presenting with rectal prolapse. The abstract of this study was presented as a poster at the American College of Gastroenterology Annual Scientific Meeting on 24 October 2021.

## Case presentation

**Figure 1:**
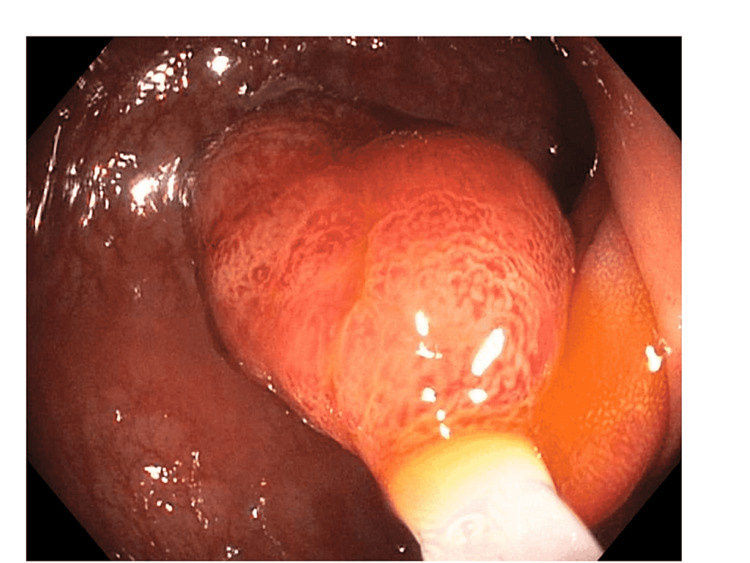
Endoscopic image in the distal sigmoid colon This image demonstrates a 20 mm-pedunculated polyp with hyperemic mucosa and a long stalk.

A 44-year-old male with no significant past medical history presented to the emergency department with rectal prolapse and bleeding that developed while straining to have a bowel movement. His mother was diagnosed with colon cancer in her 50s. Physical examination was consistent with a complete external rectal prolapse. A complete blood count (CBC) was unremarkable with a hemoglobin of 16 g/dL. Computed tomography of abdomen and pelvis confirmed rectal prolapse, but could not exclude an underlying mass. The prolapsed rectum was reduced, and a colonoscopy was recommended. During the colonoscopy, a 20 mm-pedunculated polyp (Paris class Ip) with hyperemic mucosa and a long stalk was found in the distal sigmoid colon (Figure 1).

**Figure 2:**
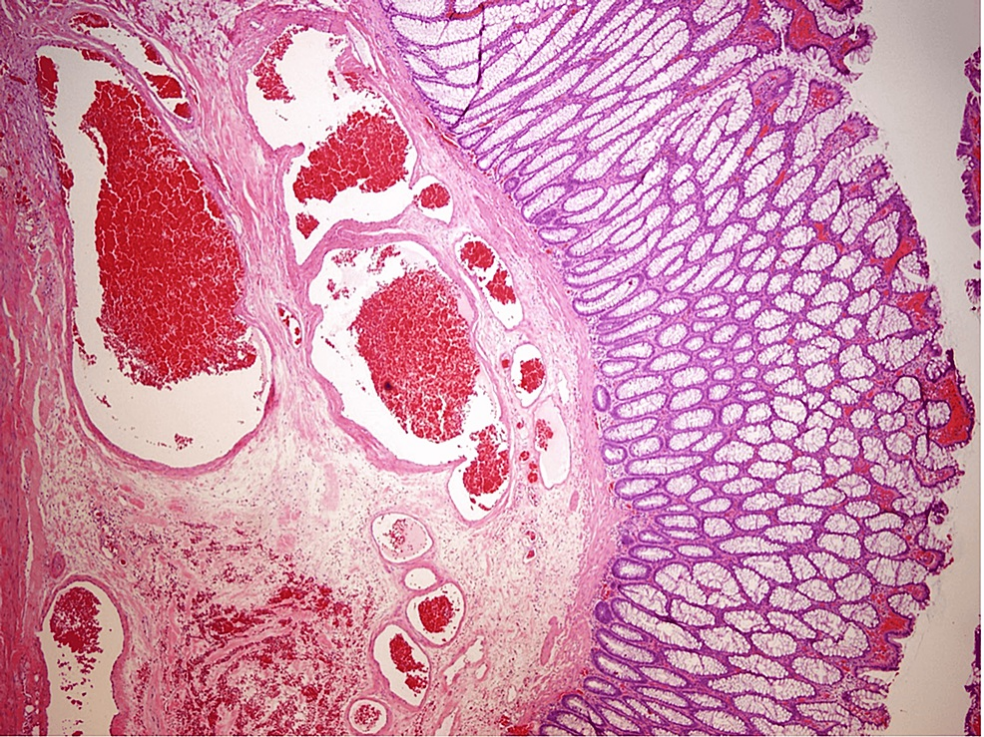
Histopathologic examination image Low-power photograph showing colonic type mucosa with surface erosion, reactive changes, and underlying submucosal dilated vascular spaces (vascular ectasia) in addition to a large thrombosed vascular space. 1X magnification.

**Figure 3:**
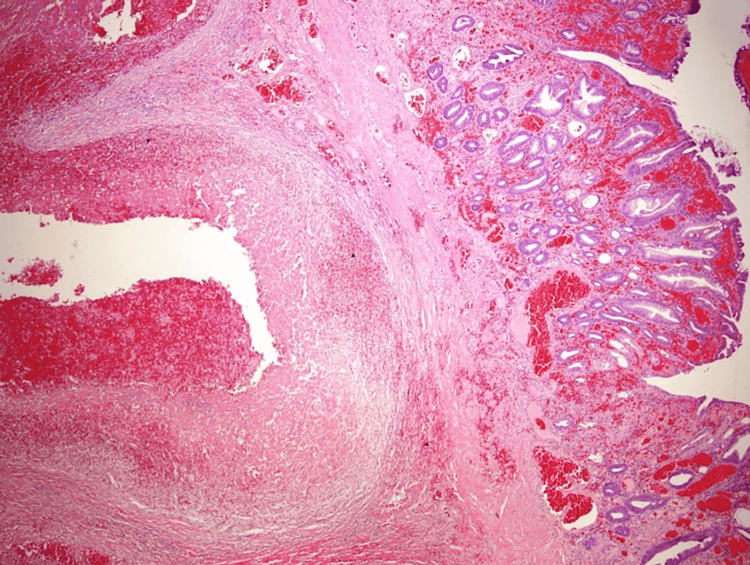
Histopathologic examination image Low-power photograph showing colonic type mucosa with underlying submucosal abnormally dilated congested vascular spaces consistent with vascular ectasia. 1X magnification.

The polyp was removed with hot-snare polypectomy without immediate or delayed complications. Histopathologic examination showed colonic mucosa with erosion, reactive epithelial change, and underlying submucosal dilated vessels with extensive thrombosis (Figures 2, 3). The diagnosis of colonic vascular ectasia presenting as a pedunculated polyp was made.

## Discussion

The incidence of VE discovered on colonoscopy was reported as 1-6% [1]. Morphologically, VE are composed of dilated thin-walled vessels which are lined by endothelium only or endothelium along with small amounts of smooth muscle [2]. Histologically, VE represent dilated vessels covered by surface epithelium [2]. These dilated vessels can occur in the mucosa or submucosa which leads to the creation of a mucosal or submucosal vascular ectasia. The pathogenesis of these lesions are still not well understood. The leading proposed mechanism is that these lesions develop with aging due to intermittent and recurrent obstruction of mucosal and submucosal veins as a result of increased contractility at the level of the muscularis propria [3-4]. The recurrent obstruction results in increased pressure and congestion in venules and capillaries thus causing vascular dilatation and arteriovenous shunts [3-4]. Endoscopically typical mucosal and submucosal vascular ectasia present as a flat or slightly raised fern like lesion. Vascular ectasia have also been found to occur in association with a variety of systemic diseases such as renal failure, cirrhosis and aortic stenosis [5]. Endoscopically a submucosal vascular ectasia presenting as a pedunculated polypoid lesion is rare.

Of the 16 previously reported VEPPL cases, these lesions were typically removed endoscopically via snare polypectomy or mucosal resection. During one reported case, the polyp was removed with snare polypectomy that resulted in excessive bleeding and required injection of epinephrine and argon plasma coagulation [6-7]. The other cases of snare polypectomy were successful without complication. In one reported case, the lesion required snare polypectomy with endoscopic mucosal resection [8]. While another case required bowel resection because of the lesion's large size that was causing intussusception [8-9]. In our case, the patient's polyp was successfully removed via hot snare without complication. The patient has not experienced reoccurrence of rectal prolapse since the procedure.

Typical flat vascular ectasia lesions are most commonly found in the cecum and ascending colon [5]. Interestingly, VEPPL were most commonly found within the sigmoid colon [10]. In seven cases, VEPPL lesions were found within the sigmoid colon [10]. Three cases reported VEPPL within the transverse colon [10]. VEPPL was reported within the cecum in two cases [10]. While the ascending colon, descending colon and rectum had one case of VEPPL reported respectively [10]. In our case, the VEPPL was discovered within the sigmoid colon.

## Conclusions

In contrast to flat or slightly raised classical vascular ectasia which are reported in 1-6% of colonoscopies, submucosal vascular ectasias presenting as pedunculated polypoid lesions are rare and have only been described in 16 other cases in the literature. Endoscopically, vascular ectasia pedunculated polypoid lesions can appear similarl to adenomatous polyps which makes it difficult to diagnose these lesions during colonoscopy. Other cases have treated this pedunculated polyp with polypectomy. The most common location of VEPPL was within the sigmoid colon. In conclusion, while all other reported cases of submucosal vascular ectasia pedunculated polypoid lesions were either asymptomatic or presented with lower gastrointestinal bleeding or iron deficiency anemia, we present a rare case of a submucosal vascular ectasia pedunculated polypoid lesion with a unique presentation of rectal prolapse.
